# Prevalence of Antibiotic Resistome in Ready-to-Eat Salad

**DOI:** 10.3389/fpubh.2020.00092

**Published:** 2020-03-25

**Authors:** Shu-Yi-Dan Zhou, Meng-Yun Wei, Madeline Giles, Roy Neilson, Fei Zheng, Qi Zhang, Yong-Guan Zhu, Xiao-Ru Yang

**Affiliations:** ^1^Key Laboratory of Urban Environment and Health, Institute of Urban Environment, Chinese Academy of Sciences, Xiamen, China; ^2^University of the Chinese Academy of Sciences, Beijing, China; ^3^Ecological Sciences, The James Hutton Institute, Dundee, United Kingdom; ^4^College of Environment, Zhejiang University of Technology, Hangzhou, China; ^5^Center for Excellence in Regional Atmospheric Environment, Institute of Urban Environment, Chinese Academy of Sciences, Xiamen, China

**Keywords:** ready-to-eat food, salad, antibiotic resistance genes (ARGs), high-throughput quantitative PCR, human health

## Abstract

Ready-to-eat salad harbors microorganisms that may carry various antibiotic resistance genes (ARGs). However, few studies have focused on the prevalence of ARGs on salad, thus underestimating the risk of ARGs transferring from salad to consumers. In this small-scale study, high-throughput quantitative PCR was used to explore the presence, prevalence and abundance of ARGs associated with serving salad sourced from two restaurant types, fast-food chain and independent casual dining. A total of 156 unique ARGs and nine mobile genetic elements (MGEs) were detected on the salad items assessed. The abundance of ARGs and MGEs were significantly higher in independent casual dining than fast-food chain restaurants. Absolute copies of ARGs in salad were 1.34 × 10^7^ to 2.71 × 10^8^ and 1.90 × 10^8^ to 4.87 × 10^8^ copies per g salad in fast-food and casual dining restaurants, respectively. Proteobacteria, Bacteroidetes, Actinobacteria, and Firmicutes were the dominant bacterial phyla detected from salad samples. *Pseudomonas, Acinetobacter, Exiguobacterium, Weissella, Enterobacter, Leuconostoc, Pantoea, Serratia, Erwinia*, and *Ewingella* were the 10 most dominant bacterial genera found in salad samples. A significant positive correlation between ARGs and MGEs was detected. These results integrate knowledge about the ARGs in ready-to-eat salad and highlight the potential impact of ARGs transfer to consumers.

## Introduction

Antibiotic resistance genes (ARGs) are widely distributed in the natural world (1). In a food production context, the phyllosphere of plants and vegetables are regarded as a reservoir of ARGs and antibiotic resistant bacteria (ARB) (2). ARGs have been shown to subsequently transfer to human pathogens and commensal bacteria by horizontal gene transfer (HGT) leading to potential serious infection (3–5). Multiple pathways of potential contamination between farm to fork exist for ready-to-eat salad products (6–9). Consequently, the potential risk of ARGs transferring from raw vegetables to consumers is viable (10–12). The application of antibiotics in livestock production and subsequent use of livestock manure as an organic fertilizer in vegetable production is common practice to promote the health and growth of livestock and vegetable yields (13, 14). This recycling and subsequent use of livestock manure and also sludge from wastewater treatment plants is a cost-effective alternative to mineral fertilizers (15). However, such alternative fertilizers are considered as a reservoir of ARGs and ARB (16–19) through the incomplete metabolism of antibiotics. It has been shown that the abundance of ARGs in both soil and phyllosphere can increase through application of manure-derived fertilizers (20–23). Thus, the presence of ARGs and mobile genetic elements (MGEs) can drive selection pressure of antibiotic resistant bacteria within microbial communities in the wider environment (24). Thereby, generating a pathway for ARGs and ARB to be transferred into the human food chain from produce such as raw salad that typically lacks processing (25, 26).

Furthermore, irrigation water applied to arable and horticultural crops is also a known pathway of ARGs and MGEs to the phyllosphere of edible crops and the wider environment through runoff (27–29). Once harvested, opportunities for (cross-)contamination of produce with ARGs exist during transportation (30). Single use or recycled containers for transporting raw vegetables and fruit have been reported to promote the growth of pathogen biofilms that can also harbor members of the resistome through insufficient cleaning and sanitization (31–33).

Notwithstanding the opportunity for contamination during the production and logistic phases of the supply chain (34), thorough washing of the raw produce in restaurants immediately prior to serving to the consumer is a key mitigating strategy to minimize ARG and ARB spread (35). Thus, the use of appropriate hygiene and sanitization practices are crucial, as is the health status, training and personal hygiene of individuals involved in preparation of raw food such as salad (36, 37).

With the global pursuit of a healthy lifestyle, consumption of ready-to-eat salad has considerably increased the potential exposure of the consumer to ARGs and ARBs (22). Whilst, previous studies have focused on the isolation of pathogens that contain ARGs from ready-to-eat salad (38, 39), in contrast, detection of ARGs and MGEs has been limited and thus have likely underestimated the diversity and abundance of ARGs associated with salad. A more comprehensive overview is therefore necessary to understand the distribution and prevalence of ARGs in readily available salad from typical dining outlets such as fast-food and casual dining.

This small-scale study was conducted to ascertain the presence and prevalence of ARGs in fresh ready-to-eat salad items purchased from fast-food chain and independent casual dining restaurants. By using high-throughput quantitative PCR (HT-qPCR) and 16S rRNA sequencing, this study aimed to (1) characterize and quantify ARGs and (2) compare the composition ARGs and MGEs communities between fast-food chain and casual dining restaurants.

## Materials and Methods

### Sample Collection

Samples of fresh ready-to-eat salad were obtained from 10 restaurants of two types namely; globally recognized fast-food chain (*n* = 5) and independently owned and managed casual dining (*n* = 5), located in Xiamen city, China (24°48′ N, 118°08′ E) during spring of 2019. In the fast-food chain cohort, three of the restaurants were part of the same well-known global brand. The casual dining restaurants were chosen on the basis of those serving the same salad produce as fast-food chain restaurants. Prior to entering each restaurant to collect samples, individuals used hand sanitizers to minimize sample contamination. From each restaurant, three random servings of mixed fresh ready-to-eat vegetable salad were purchased (three samples each time, 15 servings from each category and 30 in total). These three servings per restaurant were mixed as one representative sample of each category and mean abundance of antibiotic resistome used for relative comparisons. Salad samples were ordered with no additions, e.g., condiments, and stored under aseptic conditions as soon as practical at 4°C within 24 h prior to sample processing.

### DNA Extraction

DNA was extracted from salad items as previously described (40) but with the following modifications: ~25 g salad leaf from each replicate sample was weighed and placed into individual conical flasks (250 ml) containing 100 ml 0.01 M sterile phosphate buffered saline (PBS) and mixed. Prior to shaking (180 rpm) at 30°C for 1.5 h, samples were sonicated for 10 min. Any residual salad leaf was discarded, though this may have resulted in a small amount of adhering bacteria being lost, and the washing solution filtered through a nylon gauze and then further filtered with a cellulose membrane filter (0.22 μm), which was cut into pieces with sterilized scissors and subjected to DNA extraction using a FastDNA spin Kit for soil (MP Biomedical, Santa Ana, CA) following the manufacturer's instruction. Quality and concentration of DNA were assessed by a NanoDrop ND-1000 (Nanodrop ND-1000, Thermo Scientific, Waltham, MA) and QuantiFluor dsDNA system (Promega, Madison, WI), respectively, following protocols provided by the manufacturers. DNA concentration of each sample was equilibrated at 20 ng μl^−1^.

### High-Throughput Quantitative PCR (HT-qPCR)

In this study, high-throughput quantitative PCR (HT-qPCR) was performed by using the Wafergen SmartChip Real-time PCR system (18, 41). Primer sets for 283 ARGs, 12 MGEs (8 transposases, 1 clinic integron, 1 class 1 integron, 1 class 2 integron, and 1 class 3 integron) and 1 16S rRNA gene, were used (Table S1). HT-qPCR PCR conditions were as previously described (18), with an initial enzyme activation at 95°C for 10 min thereafter 40 amplification cycles as follows: denaturation at 95°C for 30 s and annealing at 60°C for 30 s. The detection limit was set at a threshold cycle of 31 (C_T_) and amplification was only considered as positive if all three technical replicates showed a positive result. Relative gene copy number and a normalized gene copy number per bacteria were calculated as follows:

$$\begin{array}{l} \text{Relative gene copy number }={\text{10}}^{\left(\left({\text{31-C}}_{\text{T}}\right)\text{/}\left(\text{10/3}\right)\right)}\\ \text{Normalized ARG copy number }=(\text{Relative ARG copy number/}\\ \text{ Relative 16S rRNA gene copynumber})\;\times \text{ 4}.\text{1} \end{array}$$

where C_T_ is the threshold value, and 4.1 is the average number of 16S rRNA genes relative to a bacterial cell based on the Ribosomal RNA Operon Copy Number Database (42). The 16S rRNA gene copy numbers used for calculation of absolute copy numbers were quantified by the Wafergen chip platform (40).

### Illumina Sequencing of 16S rRNA

The V4-V5 region of 16S rRNA was amplified with the universal primers 515F: GTGCCAGCMGCCGCGG and 907R: CCGTCAATCMTTTRAGTTT (43). To identify each sample, a unique barcode was applied (44). Initial enzyme activation was at 95°C for 5 min followed by 35 amplification cycles of 30 s at 94°C, 35 s at 58°C and 30 s at 72°C (40). Sequencing of these barcoded amplicons was performed using an Illumina Hiseq 2500 platform (Novogene, Beijing, China).

Bioinformatic analyses was performed using USEARCH (10.0.240) (http://www.drive5.com/usearch/). Based on UPARSE clustering, reads were separated into Zero-Operational taxonomic units (ZOTUs). ZOTUs were set at 100% similarity level and chimeras filtered with UNOISE2 (45). ZOTUs represented by a single sequence were discarded. The Ribosomal Database Project, which uses the Greengenes database (Version 13.8, 16S rRNA gene database), was used to assign the relative abundance and taxonomic identity of ZOTUs (46, 47). The data generated in this study were deposited in the NCBI Sequence Read Archive (SRA) under the accession number PRJNA552669.

### Statistical Analysis

Pearson Correlation Analysis, student's *T*-test and Analysis of Variance (ANOVA) were conducted using SPSS (v21) Box chart, Ordinary Least-Squares (OLS) and Non-metric Multidimensional scaling (NMDS) analyses were conducted with the R package, “Vegan” (48) and visualized using “ggpolt2” version 3.1 (49). Bar- and pie-charts were created by Originlab 2018. Dominant bacterial genera in each sample were visualized using Circos software (50). Basic analysis of raw data, including the calculation of mean and standard errors, was conducted in MS Excel 2016.

## Results

### Diversity and Abundance of ARGs and MGEs

**Figure 1:**
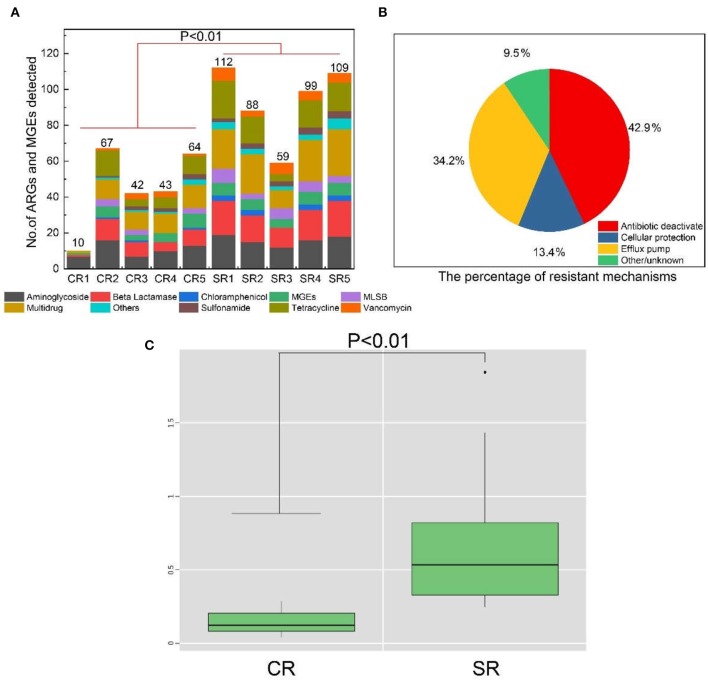
Number of ARGs and MGEs detected in fast-food chain (CR) and casual dining restaurants (SR) **(A)**. Percentage of antibiotic resistance mechanisms detected in ARGs and MGEs from fast-food chain (CR) and casual dining (SR) restaurants **(B)**. Normalized abundance of ARGs in fast-food chain (CR) and casual dining (SR) restaurants **(C)**. CR1-CR5 and SR1-SR5 are sample codes pertaining to the samples of salad collected from each restaurant type.

The ARGs detected from salad samples comprised of nine recognized classes: aminoglycoside, Beta-Lactamase, chloramphenicol, Macrolide-Lincosamide-Streptogramin B (MLSB), multidrug, sulfonamide, tetracycline, vancomycin and others based on the antibiotic type (Figure 1A). Overall, a total of 219 ARGs and nine MGEs (6 transposases, 1 class 1 integron, 1 clinic integron and 1 class 2 integron) were detected. A significantly higher prevalence of ARGs and MGEs was detected in samples from casual dining compared to fast-food restaurants (Figure S1) (*P* < 0.01) (ANOVA). The detected number of ARGs and MGEs across all salad samples ranged from 10 to 112 (Figure 1A) with a greater (*P* < 0.01) number of ARGs and MGEs from casual dining compared to fast-food restaurants. Four antibiotic resistance mechanisms were identified from samples as follows: antibiotic deactivate (42.9%), efflux pump (34.2%), other /unknown (9.5%) and cellular protection (13.4%) (Figure 1B).

**Figure 2:**
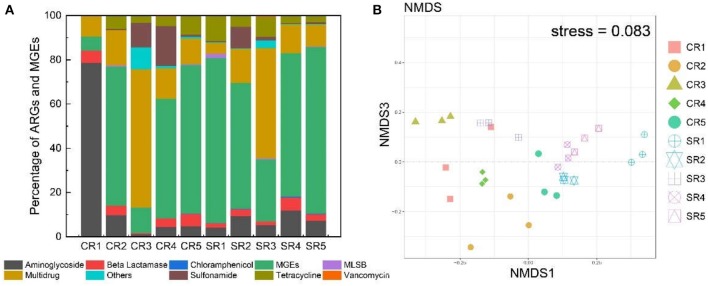
Percentage of ARGs and MGEs based on the overall abundance of ARGs and MGEs in each sample **(A)**. Non-metric Multidimensional scaling (NMDS) analysis of ARGs from all samples **(B)**. The two-dimensional stress was 0.083. Different colors and shapes represent the different samples.

The normalized abundance of ARGs from salad samples collected from casual dining restaurants was significantly greater (*P* < 0.01) than those from fast-food restaurants (Figure 1C). The absolute abundance of ARGs in samples from fast-food and casual dining restaurants ranged from 1.34 × 10^7^ (sample CR5) to 2.71 × 10^8^ (CR3) and 1.90 × 10^8^ (SR5) to 3.08 × 10^9^ copy number g^−1^ (SR1), respectively (Figure S2). The absolute abundance of MGEs from fast-food restaurants and casual dining restaurants ranged from 3.17 × 10^6^ (CR4) to 9.46 × 10^7^ (CR2) and 4.67 × 10^7^ (SR3) to 4.88 × 10^9^ (SR1), respectively (Figure S2). Aminoglycoside accounted for >75% of ARGs in sample CR1 (Figure 2A) with multidrug dominant in CR3 and SR3, respectively. MGEs, which consisted of transposases and integron genes, were the most abundant group among the remaining samples (Figure 2A). With the exception of sample CR1, multidrug and MGEs contributed >70% of the total ARGs (Figure 2A).

The diversity of ARGs and MGEs showed different patterns in samples of CR3, CR4, and CR5 (Figure 2B), which belonged to the same fast-food brand of restaurant but were geographically separated by at least 10 km across Xiamen city. For these three samples, abundance of tetracycline in CR5 was significantly >CR3 and CR4 (*P* < 0.05, ANOVA). In contrast, sulfonamide abundance was higher in CR3 and CR4 than CR5 (*P* < 0.05, ANOVA) (Figure 2A). Nonmetric Multidimensional scaling (NMDS) analysis (stress = 0.083) revealed that, in general, community compositions of samples were distinct from each other (Figure 2B).

### Correlation Between ARGs and MGEs

**Figure 3:**
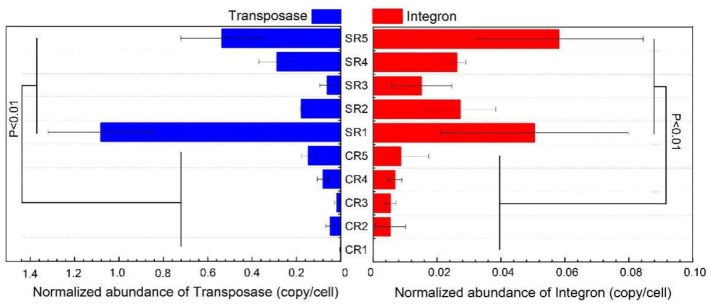
Normalized abundance of transposase and integron from sampled fast-food chain (CR) and casual dining (SR) restaurants.

Integron and transposases genes were detected in salad samples collected from all restaurants except for sample CR1 where integron genes were not recorded (Table 1). Integron genes, including *intI-1LC, intI-1(clinic)*, and *intI2*, were detected in four out of 10 sampled restaurants (CR5, SR1, SR4, SR5; Table 1). Transposase gene *tnpA-01* was ubiquitous in all samples except SR1, and *tnpA-03 and tnpA-05* genes were common to all samples, except CR1 and CR3 (Table 1). The normalized abundance of both integron and transposase genes across all sampled restaurants ranged from 0 to 0.057 and 0.0022 to 1.07 copies per cell, respectively and for both integron and transposase genes normalized abundance was significantly greater (*P* < 0.01) in samples from casual dining than those from fast-food restaurants (Figure 3).

**Table 1:** Occurrence of integron and transposase genes from each sampled restaurant.

**Sample sites**	**Integron genes**	**Transposase genes**
CR1		*tnpA-01*
CR2	*intI-1LC, intI-1(clinic)*	*tnpA-01,tnpA-03, tnpA-04, tnpA-05*
CR3	*intI-1LC*	*tnpA-01, tnpA-02*.
CR4	*intI-1LC*	*tnpA-01,tnpA-02, tnpA-03, tnpA-05*
CR5	*intI-1LC, intI-1(clinic), intI2*	*tnpA-01,tnpA-02, tnpA-03, tnpA-04, tnpA-05*
SR1	*intI-1LC, intI-1(clinic), intI2*	*tnpA-03, tnpA-04, tnpA-05, tnpA-07*
SR2	*intI-1LC, intI-1(clinic)*	*tnpA-01, tnpA-03, tnpA-05, tnpA-07*
SR3	*intI-1LC, intI-1(clinic)*	*tnpA-01, tnpA-03, tnpA-05*
SR4	*intI-1LC, intI-1(clinic), intI2*	*tnpA-01,tnpA-02, tnpA-03, tnpA-05*
SR5	*intI-1LC, intI-1(clinic), intI2*	*tnpA-01,tnpA-02, tnpA-03, tnpA-05*

**Figure 4:**
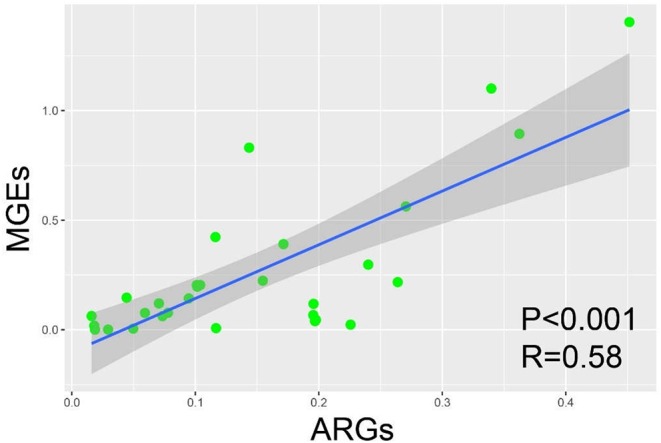
Ordinary least-squares (OLS) analysis between ARGs and MGEs among the samples from fast-food chain and casual dining restaurants.

A significant positive correlation existed between ARGs and MGEs (*P* < 0.001, *R*^2^ = 0.58; Ordinary least-squares (OLS) analysis; Figure 4). Normalized abundance of MGEs was positively correlated (*P* < 0.01; Pearson correlation analysis) with abundance of Aminoglycoside, MLSB and Tetracycline resistant genes (Table S2).

### Characterization of Bacterial Communities and Correlation With ARGs

**Figure 5:**
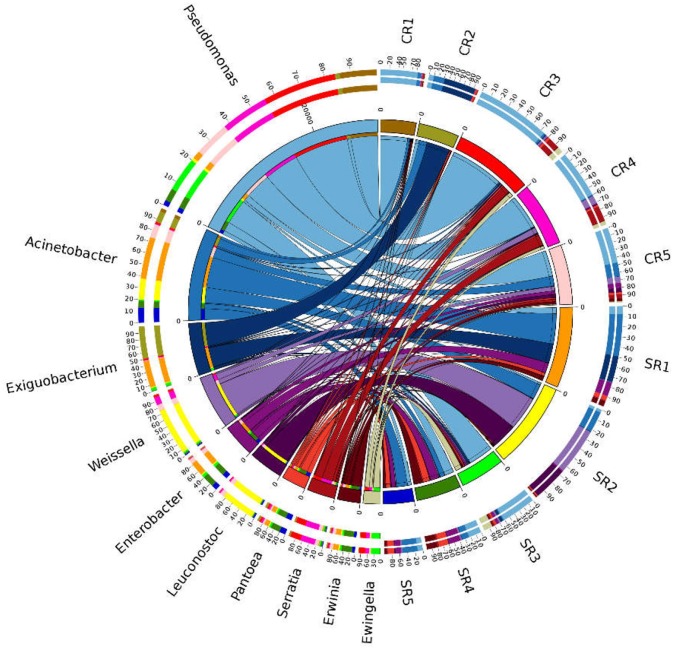
Composition of the 10 most prevalent bacterial genera detected from all samples. Data were depicted by Circos software. Length of the bars (sites) represent the percentage of the respective genus from each sample. Length of the bars (genus) represents the percentage the sample contributes to the proportion of each bacterial genus.

A total of 7,83,077 high quality sequences were detected (ranging from 2,636 to 18,377 sequences per sample), and clustered into 366 distinct ZOTUs (ranging from 128 to 287 ZOTUs per sample). Overall, only four phyla Proteobacteria, Bacteroidetes, Actinobacteria and Firmicutes, were detected (Figure S3). The 10 most dominant bacterial genera detected in salad samples were *Pseudomonas, Acinetobacter, Exiguobacterium, Weissella, Enterobacter, Leuconostoc, Pantoea, Serratia, Erwinia, and Ewingella* (Figure 5). *Pseudomonas* were dominant in restaurants CR1, CR3, CR4, CR5, and SR3, *Acinetobacter* dominated in SR1, *Exiguobacterium* dominated in CR2, and *Weissella* dominated in SR2 (Figure 5).

The proportion of *Pseudomonas* (*P* = 0.002, *t* = 3.323) and *Serratia* (*P* = 0.007, *t* = 3.138) in fast-food restaurants was significantly greater than casual dining restaurants. In contrast, a significantly higher proportion of *Enterobacter* (*P* = 0.009, *t* = −2.927), *Erwinia* (*P* = 0.018, *t* = −2.646) and *Pantoea* (*P* = 0.038, *t* = −2.276) was detected in casual dining than fast-food restaurants.

A correlation existed between ARGs and the composition (ZOTUs) of bacterial communities on salad samples (Procrustes sum of squares *M*^2^ = 0.65, *r* = 0.59, *P* < 0.001, 9999 free permutations) (Figure S4).

## Discussion

### Prevalence of ARGs in Salad

Notwithstanding the small-scale nature of the study, to our knowledge this is the first analysis of ARGs in salad by HT-qPCR. Previous reports have indicated the presence of various ARGs associated with the phyllosphere of in-field vegetables (51–53) as well as harvested and processed ready-to-eat vegetables (8, 54). Unlike the HT-qPCR, conventional PCR is not robust enough to detect many ARGs in a single reaction, which may result in finite detection of ARGs and thus an underestimation of the prevalence of ARGs throughout the vegetable supply chain (55).

A total of 156 unique ARGs representing four major antibiotic resistant mechanisms (antibiotic deactivation, efflux pumping, cellular protection, and other unknown) were detected in salad samples assessed, irrespective of restaurant type. In a human health context, consumers are thus potentially being exposed to ARGs directly from ready-to-eat salad. Few studies have to date focused on the abundance of ARGs that may be ingested directly through consumption of salad. However, we acknowledge that culture techniques were not used in this study, and thus we were unable to determine the proportion of detected ARGs/MGEs that were sourced from viable bacteria on the sampled salad. Based on our sample size, consumers are typically exposed to 1.34 × 10^7^ to 2.71 × 10^8^ and 1.90 × 10^8^ to 4.87 × 10^8^ copies of ARGs g^−1^ salad total in fast-food and casual dining restaurants, respectively. Since no hygiene standard has previously been established based on prevalence and abundance of ARGs, the absolute abundances reported here provide potential initial reference data to develop a baseline to measure efficacy of current and future hygiene standards.

Although previous studies have identified hygiene protocols to eliminate ARGs from the raw and ready-to-eat vegetables supply chain (22, 56), our results suggest that either hygiene protocols are not being adhered to or are inefficient. The significantly greater normalized abundance (*P* < 0.01) of ARGs in independently owned and managed casual dining restaurants may be attributed to a lack of standardized hygiene protocols for washing and preparing ready-to-eat salad. Although the use of hygiene protocols along the supply chain could mitigate the abundance of ARGs to some extent, environmental conditions such as temperature and humidity during transportation and storage may also be key factors affecting the preservation and transfer of ARGs (30). In agricultural production systems, the use of antibiotics, application of organic fertilizers and irrigating with reclaimed water is known to enrich ARGs in the wider environment (57–60). Thus, reduced antibiotic burden is required to limit the exposure and distribution of antibiotics (61). Moreover, a switch from antibiotic contaminated organic fertilizers to pretreated composts and amendments, e.g., biochar should be considered (62). Also, technologies such as ozone treatment may be used to reduce microbial growth and mitigate the risk of ARG infected pathogens (63). In the food preparation environment, contamination of salad by utensils and surfaces used to prepare fish and meat are well-documented (64, 65). Furthermore, the health and working practices of individuals involved in food preparation are also recognized as a means of transferring ARGs to food items (66). The observed positive significant correlation between the phyllosphere microbiome and ARGs in salad samples of this study, suggesting that the microbial community composition may contribute to the movement and subsequent transfer of ARGs, concurring with a previous study (67).

### MGEs Profile

A total of six transposase and three integron genes were detected with the abundance of integron (*P* < 0.01, ANOVA) and transposase (*P* < 0.01, ANOVA) genes significantly greater in casual dining than fast-food restaurants. Based on our sample size (300 g salad), consumers were potentially exposed to 9.51 × 10^8^ to 2.84 × 10^10^ (fast-food) and 1.40 × 10^10^ to 1.46 × 10^12^ (casual dining) absolute copies of MGEs per salad serving. Similar to ARGs, data generated here could be used as a reference to assess hygiene standards. The significant positive correlation between MGE and ARG abundance reflects the potential for MGEs to be a driver of ARG abundance. It is of note that in our study, the abundance of MGEs and anti-Multidrug genes dominated the ARGs in samples from most restaurants. Therefore, it is clear that more stringent hygiene protocols are required for processing of salad items (56).

In conclusion, we found that ARGs and MGEs were prevalent in ready-to-eat salad sourced from both fast-food chain or casual dining restaurants with the abundance of ARGs and MGEs significantly higher in casual dining than fast-food chain restaurants. Furthermore, we noted a significant positive correlation between ARGs and MGEs. Previously published data (8) has reported that transferable members of the resistome were found in salad or ready-to-eat vegetables. However, the multiple occurrences of diverse communities of ARGs and MGEs in ready-to-eat salad reported in this study has highlighted the level of exposure to consumers that has potential for downstream transfer to human gut pathogens or commensals (68, 69). We fully recognize that to build a public health framework to manage the potential impact of ARGs to consumers, a more comprehensive and scaled sampling strategy than deployed in this small-scale study is required. Furthermore, standardized best practice methodologies would need to be developed, validated including a ring-testing step and deployed at city/regional/national scale. Notwithstanding this, this study demonstrated on a small-scale using HT-qPCR that ARGs and MGEs are present and prevalent in two popular dining experiences (fast-food and casual dining) with varying abundance. As such, this study highlighted a potential public health issue that is congruent with wider global concerns on antimicrobial resistance.

## Data Availability Statement

The datasets generated for this study can be found in the NCBI Sequence Read Archive (SRA), SUB5898138.

## Author Contributions

S-Y-DZ, M-YW, and X-RY designed the experimental protocol and carried out the experiments. MG, RN, FZ, QZ, and Y-GZ assisted with the experiments. S-Y-DZ and M-YW wrote the manuscript. MG, RN, Y-GZ, and X-RY revised the manuscript. All authors have read and approved the manuscript.

### Conflict of Interest

The authors declare that the research was conducted in the absence of any commercial or financial relationships that could be construed as a potential conflict of interest.
