# Supplementary material for: Prevalence of Antibiotic Resistome in Ready-to-Eat Salad
Source: Front Public Health. 2020 Mar 25;8:92. doi: 10.3389/fpubh.2020.00092 (PMC7109403; doi:10.3389/fpubh.2020.00092)
Supplement: Supplementary file 1 [file Data_Sheet_1.docx]

**Prevalence of antibiotic resistome in ready-to-eat salad**

Shu-Yi-Dan Zhou^1,2#^, Meng-Yun Wei^1,2#^, Madeline Giles^3^, Roy Neilson ^3^,Fei Zheng^1,2^, Qi Zhang^4^, Yong-Guan Zhu^1,2,5^, Xiao-Ru Yang^1*^

*^1^Key Laboratory of Urban Environment and Health, Institute of Urban Environment, Chinese Academy of Sciences, 1799 Jimei Road, Xiamen 361021, China*

*^2^University of the Chinese Academy of Sciences, 19A Yuquan Road, Beijing 100049, China*

*^3^**Ecological Sciences, The James Hutton Institute, Dundee, DD2 5DA, Scotland, UK*

*^4^College of Environment, Zhejiang University of Technology, Hangzhou 310032, P. R. of China*

*^5^State Key Laboratory of Urban and Regional Ecology, Research Center for Eco-Environmental Sciences, Chinese Academy of Sciences, Beijing 100085, China*

**Contents**

**Tables:**

Table S1. List of primers used for qPCR with high through put chip this study.

Table S2. Pearson correlation analysis between MGEs and classes of ARGs.

**Figures**:

Figure S1. Heatmap analysis of ARGs and MGEs in salad samples.

Figure S2. Absolute copy numbers of ARGs and MGEs in salad samples from fast-food (CR) chain and casual dining (SR) restaurants.

Figure S3. Percentage of detected phyla in salad samples.

Figure S4. Procrustes analysis between ZOTUs and ARGs profiles

**Total:**

**Number of tables: 2**

**Number of figures: 4**

**Number of pages: 20**

Table S1. List of primers used for qPCR with high through put chip this study.

| 16S rRNA | GGGTTGCGCTCGTTGC | ATGGYTGTCGTCAGCTCGTG | 16S rRNA |
| --- | --- | --- | --- |
| aac | CCCTGCGTTGTGGCTATGT | TTGGCCACGCCAATCC | Aminoglycoside |
| aac(6')I1 | GACCGGATTAAGGCCGATG | CTTGCCTTGATATTCAGTTTTTATAACCA | Aminoglycoside |
| aac(6')-Ib(aka aacA4)-01 | GTTTGAGAGGCAAGGTACCGTAA | GAATGCCTGGCGTGTTTGA | Aminoglycoside |
| aac(6')-Ib(aka aacA4)-02 | CGTCGCCGAGCAACTTG | CGGTACCTTGCCTCTCAAACC | Aminoglycoside |
| aac(6')-Ib(aka aacA4)-03 | AGAAGCACGCCCGACACTT | GCTCTCCATTCAGCATTGCA | Aminoglycoside |
| aac(6')-II | CGACCCGACTCCGAACAA | GCACGAATCCTGCCTTCTCA | Aminoglycoside |
| aac(6')-Iy | GCTTTGCGGATGCCTCAAT | GGAGAACAAAAATACCTTCAAGGAAA | Aminoglycoside |
| aacA/aphD | AGAGCCTTGGGAAGATGAAGTTT | TTGATCCATACCATAGACTATCTCATCA | Aminoglycoside |
| aacC | CGTCACTTATTCGATGCCCTTAC | GTCGGGCGCGGCATA | Aminoglycoside |
| aacC1 | GGTCGTGAGTTCGGAGACGTA | GCAAGTTCCCGAGGTAATCG | Aminoglycoside |
| aacC2 | ACGGCATTCTCGATTGCTTT | CCGAGCTTCACGTAAGCATTT | Aminoglycoside |
| aacC4 | CGGCGTGGGACACGAT | AGGGAACCTTTGCCATCAACT | Aminoglycoside |
| aadA-01 | GTTGTGCACGACGACATCATT | GGCTCGAAGATACCTGCAAGAA | Aminoglycoside |
| aadA-02 | CGAGATTCTCCGCGCTGTA | GCTGCCATTCTCCAAATTGC | Aminoglycoside |
| aadA1 | AGCTAAGCGCGAACTGCAAT | TGGCTCGAAGATACCTGCAA | Aminoglycoside |
| aadA-1-01 | AAAAGCCCGAAGAGGAACTTG | CATCTTTCACAAAGATGTTGCTGTCT | Aminoglycoside |
| aadA-1-02 | CGGAATTGAAAAAACTGATCGAA | ATACCGGCTGTCCGTCATTT | Aminoglycoside |
| aadA2-01 | ACGGCTCCGCAGTGGAT | GGCCACAGTAACCAACAAATCA | Aminoglycoside |
| aadA2-02 | CTTGTCGTGCATGACGACATC | TCGAAGATACCCGCAAGAATG | Aminoglycoside |
| aadA2-03 | CAATGACATTCTTGCGGGTATC | GACCTACCAAGGCAACGCTATG | Aminoglycoside |
| aadA5-01 | ATCACGATCTTGCGATTTTGCT | CTGCGGATGGGCCTAGAAG | Aminoglycoside |
| aadA5-02 | GTTCTTGCTCTTGCTCGCATT | GATGCTCGGCAGGCAAAC | Aminoglycoside |
| aadA9-01 | CGCGGCAAGCCTATCTTG | CAAATCAGCGACCGCAGACT | Aminoglycoside |
| aadA9-02 | GGATGCACGCTTGGATGAA | CCTCTAGCGGCCGGAGTATT | Aminoglycoside |
| aadD | CCGACAACATTTCTACCATCCTT | ACCGAAGCGCTCGTCGTATA | Aminoglycoside |
| aadE | TACCTTATTGCCCTTGGAAGAGTTA | GGAACTATGTCCCTTTTAATTCTACAATCT | Aminoglycoside |
| acrA-01 | CAACGATCGGACGGGTTTC | TGGCGATGCCACCGTACT | Multidrug |
| acrA-02 | GGTCTATCACCCTACGCGCTATC | GCGCGCACGAACATACC | Multidrug |
| acrA-03 | CAGACCCGCATCGCATATT | CGACAATTTCGCGCTCATG | Multidrug |
| acrA-04 | TACTTTGCGCGCCATCTTC | CGTGCGCGAACGAACAT | Multidrug |
| acrA-05 | CGTGCGCGAACGAACA | ACTTTGCGCGCCATCTTC | Multidrug |
| acrB-01 | AGTCGGTGTTCGCCGTTAAC | CAAGGAAACGAACGCAATACC | Multidrug |
| acrF | GCGGCCAGGCACAAAA | TACGCTCTTCCCACGGTTTC | Multidrug |
| acrR-01 | GCGCTGGAGACACGACAAC | GCCTTGCTGCGAGAACAAA | Multidrug |
| acrR-02 | GATGATACCCCCTGCTGTGAGA | ACCAAACAAGAAGCGCAAGAA | Multidrug |
| adeA | CAGTTCGAGCGCCTATTTCTG | CGCCCTGACCGACCAAT | Multidrug |
| ampC/blaDHA | TGGCCGCAGCAGAAAGA | CCGTTTTATGCACCCAGGAA | Beta-lactams |
| ampC-01 | TGGCGTATCGGGTCAATGT | CTCCACGGGCCAGTTGAG | Beta-lactams |
| ampC-02 | GCAGCACGCCCCGTAA | TGTACCCATGATGCGCGTACT | Beta-lactams |
| ampC-04 | TCCGGTGACGCGACAGA | CAGCACGCCGGTGAAAGT | Beta-lactams |
| ampC-05 | CTGTTCGAGCTGGGTTCTATAAGTAAA | CAGTATCTGGTCACCGGATCGT | Beta-lactams |
| ampC-06 | CCGCTCAAGCTGGACCATAC | CCATATCCTGCACGTTGGTTT | Beta-lactams |
| ampC-09 | CAGCCGCTGATGAAAAAATATG | CAGCGAGCCCACTTCGA | Beta-lactams |
| aph | TTTCAGCAAGTGGATCATGTTAAAAT | CCAAGCTGTTTCCACTGTTTTTC | Aminoglycoside |
| aph(2')-Id-01 | TGAGCAGTATCATAAGTTGAGTGAAAAG | GACAGAACAATCAATCTCTATGGAATG | Aminoglycoside |
| aph(2')-Id-02 | TAAGGATATACCGACAGTTTTGGAAA | TTTAATCCCTCTTCATACCAATCCATA | Aminoglycoside |
| aph6ia | CCCATCCCATGTGTAAGGAAA | GCCACCGCTTCTGCTGTAC | Aminoglycoside |
| aphA1 | TGAACAAGTCTGGAAAGAAATGCA | CCTATTAATTTCCCCTCGTCAAAAA | Aminoglycoside |
| bacA-01 | CGGCTTCGTGACCTCGTT | ACAATGCGATACCAGGCAAAT | Others |
| bacA-02 | TTCCACGACACGATTAAGTCATTG | CGGCTCTTTCGGCTTCAG | Others |
| bla1 | GCAAGTTGAAGCGAAAGAAAAGA | TACCAGTATCAATCGCATATACACCTAA | Beta-lactams |
| bla-ACC-1 | CACACAGCTGATGGCTTATCTAAAA | AATAAACGCGATGGGTTCCA | Beta-lactams |
| blaCMY | CCGCGGCGAAATTAAGC | GCCACTGTTTGCCTGTCAGTT | Beta-lactams |
| blaCMY2-01 | AAAGCCTCAT GGGTGCATAAA | ATAGCTTTTGTTTGCCAGCATCA | Beta-lactams |
| blaCMY2-02 | GCGAGCAGCCTGAAGCA | CGGATGGGCTTGTCCTCTT | Beta-lactams |
| blaCTX-M-01 | GGAGGCGTGACGGCTTTT | TTCAGTGCGATCCAGACGAA | Beta-lactams |
| blaCTX-M-03 | CGATACCACCACGCCGTTA | GCATTGCCCAACGTCAGATT | Beta-lactams |
| blaCTX-M-04 | CTTGGCGTTGCGCTGAT | CGTTCATCGGCACGGTAGA | Beta-lactams |
| blaCTX-M-05 | GCGATAACGTGGCGATGAAT | GTCGAGACGGAACGTTTCGT | Beta-lactams |
| blaCTX-M-06 | CACAGTTGGTGACGTGGCTTAA | CTCCGCTGCCGGTTTTATC | Beta-lactams |
| blaGES | GCAATGTGCTCAACGTTCAAG | GTGCCTGAGTCAATTCTTTCAAAG | Beta-lactams |
| blaIMP-01 | AACACGGTTTGGTGGTTCTTGTA | GCGCTCCACAAACCAATTG | Beta-lactams |
| blaIMP-02 | AAGGCAGCATTTCCTCTCATTTT | GGATAGATCGAGAATTAAGCCACTCT | Beta-lactams |
| bla-L1 | CACCGGGTTACCAGCTGAAG | GCGAAGCTGCGCTTGTAGTC | Beta-lactams |
| blaMOX/blaCMY | CTATGTCAATGTGCCGAAGCA | GGCTTGTCCTCTTTCGAATAGC | Beta-lactams |
| blaOCH | GGCGACTTGCGCCGTAT | TTTTCTGCTCGGCCATGAG | Beta-lactams |
| blaOKP | GCCGCCATCACCATGAG | GGTGACGTTGTCACCGATCTG | Beta-lactams |
| blaOXA1/blaOXA30 | CGGATGGTTTGAAGGGTTTATTAT | TCTTGGCTTTTATGCTTGATGTTAA | Beta-lactams |
| blaOXA10-01 | CGCAATTATCGGCCTAGAAACT | TTGGCTTTCCGTCCCATTT | Beta-lactams |
| blaOXA10-02 | CGCAATTATCGGCCTAGAAACT | TTGGCTTTCCGTCCCATTT | Beta-lactams |
| blaOXY | CGTTCAGGCGGCAGGTT | GCCGCGATATAAGATTTGAGAATT | Beta-lactams |
| blaPAO | CGCCGTACAACCGGTGAT | GAAGTAATGCGGTTCTCCTTTCA | Beta-lactams |
| blaPER | TGCTGGTTGCTGTTTTTGTGA | CCTGCGCAATGATAGCTTCAT | Beta-lactams |
| blaPSE | TTGTGACCTATTCCCCTGTAATAGAA | TGCGAAGCACGCATCATC | Beta-lactams |
| blaROB | GCAAAGGCATGACGATTGC | CGCGCTGTTGTCGCTAAA | Beta-lactams |
| blaSFO | CCGCCGCCATCCAGTA | GGGCCGCCAAGATGCT | Beta-lactams |
| blaSHV-01 | TCCCATGATGAGCACCTTTAAA | TTCGTCACCGGCATCCA | Beta-lactams |
| blaSHV-02 | CTTTCCCATGATGAGCACCTTT | TCCTGCTGGCGATAGTGGAT | Beta-lactams |
| blaTEM | AGCATCTTACGGATGGCATGA | TCCTCCGATCGTTGTCAGAAGT | Beta-lactams |
| blaTLA | ACACTTTGCCATTGCTGTTTATGT | TGCAAATTTCGGCAATAATCTTT | Beta-lactams |
| blaVEB | CCCGATGCAAAGCGTTATG | GAAAGATTCCCTTTATCTATCTCAGACAA | Beta-lactams |
| blaVIM | GCACTTCTCGCGGAGATTG | CGACGGTGATGCGTACGTT | Beta-lactams |
| blaZ | GGAGATAAAGTAACAAATCCAGTTAGATATGA | TGCTTAATTTTCCATTTGCGATAAG | Beta-lactams |
| carB | GGAGTGAGGCTGACCGTAGAAG | ATCGGCGAAACGCACAAA | MLSB |
| catA1 | GGGTGAGTTTCACCAGTTTTGATT | CACCTTGTCGCCTTGCGTATA | Others |
| catB3 | GCACTCGATGCCTTCCAAAA | AGAGCCGATCCAAACGTCAT | Others |
| catB8 | CACTCGACGCCTTCCAAAG | CCGAGCCTATCCAGACATCATT | Others |
| ceoA | ATCAACACGGACCAGGACAAG | GGAAAGTCCGCTCACGATGA | Multidrug |
| cepA | AGTTGCGCAGAACAGTCCTCTT | TCGTATCTTGCCCGTCGATAAT | Beta-lactams |
| cfiA | GCAGCGTTGCTGGACACA | GTTCGGGATAAACGTGGTGACT | Beta-lactams |
| cfr | GCAAAATTCAGAGCAAGTTACGAA | AAAATGACTCCCAACCTGCTTTAT | Others |
| cfxA | TCATTCCTCGTTCAAGTTTTCAGA | TGCAGCACCAAGAGGAGATGT | Beta-lactams |
| cmeA | GCAGCAAAGAAGAAGCACCAA | AGCAGGGTAAGTAAAACTAAGTGGTAAATCT | Multidrug |
| cmlA1-01 | TAGGAAGCATCGGAACGTTGAT | CAGACCGAGCACGACTGTTG | Chloramphenicol |
| cmlA1-02 | AGGAAGCATCGGAACGTTGA | ACAGACCGAGCACGACTGTTG | Chloramphenicol |
| cmr | CGGCATCGTCAGTGGAATT | CGGTTCCGAAAAAGATGGAA | Multidrug |
| cmx(A) | GCGATCGCCATCCTCTGT | TCGACACGGAGCCTTGGT | Chloramphenicol |
| cphA-01 | GCGAGCTGCACAAGCTGAT | CGGCCCAGTCGCTCTTC | Beta-lactams |
| cphA-02 | GTGCTGATGGCGAGTTTCTG | GGTGTGGTAGTTGGTGTTGATCAC | Beta-lactams |
| dfrA1 | GGAATGGCCCTGATATTCCA | AGTCTTGCGTCCAACCAACAG | Sulfonamide |
| dfrA12 | CCTCTACCGAACCGTCACACA | GCGACAGCGTTGAAACAACTAC | Sulfonamide |
| emrD | CTCAGCAGTATGGTGGTAAGCATT | ACCAGGCGCCGAAGAAC | Multidrug |
| ereA | CCTGTGGTACGGAGAATTCATGT | ACCGCATTCGCTTTGCTT | MLSB |
| ereB | GCTTTATTTCAGGAGGCGGAAT | TTTTAAATGCCACAGCACAGAATC | Others |
| erm(34) | GCGCGTTGACGACGATTT | TGGTCATACTCGACGGCTAGAAC | MLSB |
| erm(35) | TTGAAAACGATGTTGCATTAAGTCA | TCTATAATCACAACTAACCACTTGAACGT | MLSB |
| erm(36) | GGCGGACCGACTTGCAT | TCTGCGTTGACGACGGTTAC | MLSB |
| ermA | TTGAGAAGGGATTTGCGAAAAG | ATATCCATCTCCACCATTAATAGTAAACC | MLSB |
| ermA/ermTR | ACATTTTACCAAGGAACTTGTGGAA | GTGGCATGACATAAACCTTCATCA | MLSB |
| ermB | TAAAGGGCATTTAACGACGAAACT | TTTATACCTCTGTTTGTTAGGGAATTGAA | MLSB |
| ermC | TTTGAAATCGGCTCAGGAAAA | ATGGTCTATTTCAATGGCAGTTACG | MLSB |
| ermF | CAGCTTTGGTTGAACATTTACGAA | AAATTCCTAAAATCACAACCGACAA | MLSB |
| ermJ/ermD | GGACTCGGCAATGGTCAGAA | CCCCGAAACGCAATATAATGTT | MLSB |
| ermK-01 | GTTTGATATTGGCATTGTCAGAGAAA | ACCATTGCCGAGTCCACTTT | MLSB |
| ermK-02 | GAGCCGCAAGCCCCTTT | GTGTTTCATTTGACGCGGAGTAA | MLSB |
| ermT-01 | GTTCACTAGCACTATTTTTAATGACAGAAGT | GAAGGGTGTCTTTTTAATACAATTAACGA | MLSB |
| ermT-02 | GTAAAATCCCTAGAGAATACTTTCATCCA | TGAGTGATATTTTTGAAGGGTGTCTT | MLSB |
| ermX | GCTCAGTGGTCCCCATGGT | ATCCCCCCGTCAACGTTT | MLSB |
| ermY | TTGTCTTTGAAAGTGAAGCAACAGT | TAACGCTAGAGAACGATTTGTATTGAG | MLSB |
| fabK | TTTCAGCTCAGCACTTTGGTCAT | AAGGCATCTTTTTCAGCCAGTTC | Others |
| floR | ATTGTCTTCACGGTGTCCGTTA | CCGCGATGTCGTCGAACT | Multidrug |
| folA | CGAGCAGTTCCTGCCAAAG | CCCAGTCATCCGGTTCATAATC | Sulfonamide |
| fosB | TCACTGTAACTAATGAAGCATTAGACCAT | CCATCTGGATCTGTAAAGTAAAGAGATC | Others |
| fosX | GATTAAGCCATATCACTTTAATTGTGAAAG | TCTCCTTCCATAATGCAAATCCA | Others |
| fox5 | GGTTTGCCGCTGCAGTTC | GCGGCCAGGTGACCAA | Beta-lactams |
| imiR | CCGGACTAGAGCTTCATGTAAGC | CCCACGCGGTACTCTTGTAAA | Others |
| intI1 | GCCTTGATGTTACCCGAGAG | GATCGGTCGAATGCGTGT | Integrase |
| intI-1(clinic) | CGAACGAGTGGCGGAGGGTG | TACCCGAGAGCTTGGCACCCA | Integrase |
| intI2 | TGCTTTTCCCACCCTTACC | GACGGCTACCCTCTGTTATCTC | Integrase |
| intI3 | GCCACCACTTGTTTGAGGA | GGATGTCTGTGCCTGCTTG | Integrase |
| IS613 | AGGTTCGGACTCAATGCAACA | TTCAGCACATACCGCCTTGAT | Transposase |
| lmrA-01 | TCGACGTGACCGTAGTGAACA | CGTGACTACCCAGGTGAGTTGA | MLSB |
| lnuA-01 | TGACGCTCAACACACTCAAAAA | TTCATGCTTAAGTTCCATACGTGAA | MLSB |
| lnuB-01 | TGAACATAATCCCCTCGTTTAAAGAT | TAATTGCCCTGTTTCATCGTAAATAA | MLSB |
| lnuB-02 | AAAGGAGAAGGTGACCAATACTCTGA | GGAGCTACGTCAAACAACCAGTT | MLSB |
| lnuC | TGGTCAATATAACAGATGTAAACCAGATTT | CACCCCAGCCACCATCAA | MLSB |
| marR-01 | GCGGCGTACTGGTGAAGCTA | TGCCCTGGTCGTTGATGA | Multidrug |
| matA/mel | TAGTAGGCAAGCTCGGTGTTGA | CCTGTGCTATTTTAAGCCTTGTTTCT | MLSB |
| mdetl1 | ATACAGCAGTGGATATTGGTTTAATTGT | TGCATAAGGTGAATGTTCCATGA | Multidrug |
| mdtA | CCTAACGGGCGTGACTTCA | TTCACCTGTTTCAAGGGTCAAA | MLSB |
| mdtE/yhiU | CGTCGGCGCACTCGTT | TCCAGACGTTGTACGGTAACCA | Multidrug |
| mecA | GGTTACGGACAAGGTGAAATACTGAT | TGTCTTTTAATAAGTGAGGTGCGTTAATA | Beta-lactams |
| mefA | CCGTAGCATTGGAACAGCTTTT | AAACGGAGTATAAGAGTGCTGCAA | MLSB |
| mepA | ATCGGTCGCTCTTCGTTCAC | ATAAATAGGATCGAGCTGCTGGAT | Multidrug |
| mexA | AGGACAACGCTATGCAACGAA | CCGGAAAGGGCCGAAAT | Multidrug |
| mexD | TTGCCACTGGCTTTCATGAG | CACTGCGGAGAACTGTCTGTAGA | Multidrug |
| mexE | GGTCAGCACCGACAAGGTCTAC | AGCTCGACGTACTTGAGGAACAC | Multidrug |
| mexF | CCGCGAGAAGGCCAAGA | TTGAGTTCGGCGGTGATGA | Multidrug |
| mphA-01 | CTGACGCGCTCCGTGTT | GGTGGTGCATGGCGATCT | MLSB |
| mphA-02 | TGATGACCCTGCCATCGA | TTCGCGAGCCCCTCTTC | MLSB |
| mphB | CGCAGCGCTTGATCTTGTAG | TTACTGCATCCATACGCTGCTT | MLSB |
| mphC | CGTTTGAAGTACCGAATTGGAAA | GCTGCGGGTTTGCCTGTA | MLSB |
| msrA-01 | CTGCTAACACAAGTACGATTCCAAAT | TCAAGTAAAGTTGTCTTACCTACACCATT | MLSB |
| msrC-01 | TCAGACCGGATCGGTTGTC | CCTATTTTTTGGAGTCTTCTCTCTAATGTT | MLSB |
| mtrC-01 | GGACGGGAAGATGGTCCAA | CGTAGCGTTCCGGTTCGAT | Multidrug |
| mtrC-02 | CGGAGTCCATCGACCATTTG | ATCGTCGGCAAGGAGAATCA | Multidrug |
| mtrD-02 | GGTCGGCACGCTCTTGTC | TGAAGAATTTGCGCACCACTAC | Multidrug |
| mtrD-03 | CCGCCAAGCCGATATAGACA | GGCCGGGTTGCCAAA | Multidrug |
| ndm-1 | ATTAGCCGCTGCATTGAT | CATGTCGAGATAGGAAGTG | Beta-lactams |
| nimE | TGCGCCAAGATAGGGCATA | GTCGTGAATTCGGCAGGTTTA | Others |
| nisB | GGGAGAGTTGCCGATGTTGTA | AGCCACTCGTTAAAGGGCAAT | Others |
| oleC | CCCGGAGTCGATGTTCGA | GCCGAAGACGTACACGAACAG | MLSB |
| oprD | ATGAAGTGGAGCGCCATTG | GGCCACGGCGAACTGA | Multidrug |
| oprJ | ACGAGAGTGGCGTCGACAA | AAGGCGATCTCGTTGAGGAA | Multidrug |
| pbp | CCGGTGCCATTGGTTTAGA | AAAATAGCCGCCCCAAGATT | Beta-lactams |
| pbp2x | TTTCATAAGTATCTGGACATGGAAGAA | CCAAAGGAAACTTGCTTGAGATTAG | Beta-lactams |
| Pbp5 | GGCGAACTTCTAATTAATCCTATCCA | CGCCGATGACATTCTTCTTATCTT | Beta-lactams |
| penA | AGACGGTAACGTATAACTTTTTGAAAGA | GCGTGTAGCCGGCAATG | Beta-lactams |
| pikR1 | TCGACATGCGTGACGAGATT | CCGCGAATTAGGCCAGAA | MLSB |
| pikR2 | TCGTGGGCCAGGTGAAGA | TTCCCCTTGCCGGTGAA | MLSB |
| pmrA | TTTGCAGGTTTTGTTCCTAATGC | GCAGAGCCTGATTTCTCCTTTG | Multidrug |
| pncA | GCAATCGAGGCGGTGTTC | TTGCCGCAGCCAATTCA | Others |
| putitive multidrug | AATTTTGCCGATTATTGCTGAAA | GATTGTCATCATTCGTTTATCACCAA | Multidrug |
| qac | CAATAATAACCGAAATAATAGGGACAAGTT | AATAAGTGTTCCTAGTGTTGGCCATAG | Multidrug |
| qacA | TGGCAATAGGAGCTATGGTGTTT | AAGGTAACACTATTTTCGGTCCAAATC | Multidrug |
| qacA/qacB | TTTAGGCAGCCTCGCTTCA | CCGAATCCAAATAAAACCCAATAA | Multidrug |
| qacEdelta1-01 | TCGCAACATCCGCATTAAAA | ATGGATTTCAGAACCAGAGAAAGAAA | Multidrug |
| qacEdelta1-02 | CCCCTTCCGCCGTTGT | CGACCAGACTGCATAAGCAACA | Multidrug |
| qacH-01 | GTGGCAGCTATCGCTTGGAT | CCAACGAACGCCCACAA | Multidrug |
| qacH-02 | CATCGTGCTTGTGGCAGCTA | TGAACGCCCAGAAGTCTAGTTTT | Multidrug |
| qSRA | AGGATTTCTCACGCCAGGATT | CCGCTTTCAATGAAACTGCAA | Others |
| rarD-02 | TGACGCATCGCGTGATCT | AAATTTTCTGTGGCGTCTGAATC | Multidrug |
| sat4 | GAATGGGCAAAGCATAAAAACTTG | CCGATTTTGAAACCACAATTATGATA | Others |
| sdeB | CACTACCGCTTCCGCACTTAA | TGAAAAAACGGGAAAAGTCCAT | Multidrug |
| spcN-01 | AAAAGTTCGATGAAACACGCCTAT | TCCAGTGGTAGTCCCCGAATC | Aminoglycoside |
| spcN-02 | CAGAATCTTCCTGAAAAGTTTGATGAA | CGCAGACACGCCGAATC | Aminoglycoside |
| speA | GCAAGAGGTATTTGCTCAACAAGA | CAGGGTCACCCTCATAAAGAAAA | Others |
| str | AATGAGTTTTGGAGTGTCTCAACGTA | AATCAAAACCCCTATTAAAGCCAAT | Aminoglycoside |
| strA | CCGGTGGCATTTGAGAAAAA | GTGGCTCAACCTGCGAAAAG | Aminoglycoside |
| strB | GCTCGGTCGTGAGAACAATCT | CAATTTCGGTCGCCTGGTAGT | Aminoglycoside |
| sul1 | CAGCGCTATGCGCTCAAG | ATCCCGCTGCGCTGAGT | Sulfonamide |
| sul2 | TCATCTGCCAAACTCGTCGTTA | GTCAAAGAACGCCGCAATGT | Sulfonamide |
| sulA/folP-01 | CAGGCTCGTAAATTGATAGCAGAAG | CTTTCCTTGCGAATCGCTTT | Sulfonamide |
| sulA/folP-03 | CACGGCTTCGGCTCATGT | TGCCATCCTGTGACTAGCTACGT | Sulfonamide |
| tet(32) | CCATTACTTCGGACAACGGTAGA | CAATCTCTGTGAGGGCATTTAACA | Tetracycline |
| tet(34) | CTTAGCGCAAACAGCAATCAGT | CGGTGATACAGCGCGTAAACT | Tetracycline |
| tet(35) | ACCCCATGACGTACCTGTAGAGA | CAACCCACACTGGCTACCAGTT | Tetracycline |
| tet(36)-01 | AGAATACTCAGCAGAGGTCAGTTCCT | TGGTAGGTCGATAACCCGAAAAT | Tetracycline |
| tet(36)-02 | TGCAGGAAAGACCTCCATTACAG | CTTTGTCCACACTTCCACGTACTATG | Tetracycline |
| tet(37) | GAGAACGTTGAAAAGGTGGTGAA | AACCAAGCCTGGATCAGTCTCA | Tetracycline |
| tetA-01 | GCTGTTTGTTCTGCCGGAAA | GGTTAAGTTCCTTGAACGCAAACT | Tetracycline |
| tetA-02 | CTCACCAGCCTGACCTCGAT | CACGTTGTTATAGAAGCCGCATAG | Tetracycline |
| tetB-01 | AGTGCGCTTTGGATGCTGTA | AGCCCCAGTAGCTCCTGTGA | Tetracycline |
| tetB-02 | GCCCAGTGCTGTTGTTGTCAT | TGAAAGCAAACGGCCTAAATACA | Tetracycline |
| tetC-01 | CATATCGCAATACATGCGAAAAA | AAAGCCGCGGTAAATAGCAA | Tetracycline |
| tetC-02 | ACTGGTAAGGTAAACGCCATTGTC | ATGCATAAACCAGCCATTGAGTAAG | Tetracycline |
| tetD-01 | TGCCGCGTTTGATTACACA | CACCAGTGATCCCGGAGATAA | Tetracycline |
| tetD-02 | TGTCATCGCGCTGGTGATT | CATCCGCTTCCGGGAGAT | Tetracycline |
| tetE | TTGGCGCTGTATGCAATGAT | CGACGACCTATGCGATCTGA | Tetracycline |
| tetG-01 | TCAACCATTGCCGATTCGA | TGGCCCGGCAATCATG | Tetracycline |
| tetG-02 | CATCAGCGCCGGTCTTATG | CCCCATGTAGCCGAACCA | Tetracycline |
| tetH | TTTGGGTCATCTTACCAGCATTAA | TTGCGCATTATCATCGACAGA | Tetracycline |
| tetJ | GGGTGCCGCATTAGATTACCT | TCGTCCAATGTAGAGCATCCATA | Tetracycline |
| tetK | CAGCAGTCATTGGAAAATTATCTGATTATA | CCTTGTACTAACCTACCAAAAATCAAAATA | Tetracycline |
| tetL-01 | AGCCCGATTTATTCAAGGAATTG | CAAATGCTTTCCCCCTGTTCT | Tetracycline |
| tetL-02 | ATGGTTGTAGTTGCGCGCTATAT | ATCGCTGGACCGACTCCTT | Tetracycline |
| tetM-01 | CATCATAGACACGCCAGGACATAT | CGCCATCTTTTGCAGAAATCA | Tetracycline |
| tetM-02 | TAATATTGGAGTTTTAGCTCATGTTGATG | CCTCTCTGACGTTCTAAAAGCGTATTAT | Tetracycline |
| tetO-01 | ATGTGGATACTACAACGCATGAGATT | TGCCTCCACATGATATTTTTCCT | Tetracycline |
| tetPA | AGTTGCAGATGTGTATAGTCGTAAACTATCTATT | TGCTACAAGTACGAAAACAAAACTAGAA | Tetracycline |
| tetPB-01 | ACACCTGGACACGCTGATTTT | ACCGTCTAGAACGCGGAATG | Tetracycline |
| tetPB-02 | TGATACACCTGGACACGCTGAT | CGTCCAAAACGCGGAATG | Tetracycline |
| tetPB-03 | TGGGCGACAGTAGGCTTAGAA | TGACCCTACTGAAACATTAGAAATATACCT | Tetracycline |
| tetPB-04 | AGTGGTGCAAATACTGAAAAAGTTGT | TTTGTTCCTTCGTTTTGGACAGA | Tetracycline |
| tetPB-05 | CTGAAGTGGAGCGATCATTCC | CCCTCAACGGCAGAAATAACTAA | Tetracycline |
| tetQ | CGCCTCAGAAGTAAGTTCATACACTAAG | TCGTTCATGCGGATATTATCAGAAT | Tetracycline |
| tetR-02 | CGCGATAGACGCCTTCGA | TCCTGACAACGAGCCTCCTT | Tetracycline |
| tetR-03 | CGCGATGGAGCAAAAGTACAT | AGTGAAAAACCTTGTTGGCATAAAA | Tetracycline |
| tetS | TTAAGGACAAACTTTCTGACGACATC | TGTCTCCCATTGTTCTGGTTCA | Tetracycline |
| tetT | CCATATAGAGGTTCCACCAAATCC | TGACCCTATTGGTAGTGGTTCTATTG | Tetracycline |
| tetU-01 | GTGGCAAAGCAACGGATTG | TGCGGGCTTGCAAAACTATC | Tetracycline |
| tetV | GCGGGAACGACGATGTATATC | CCGCTATCTCACGACCATGAT | Tetracycline |
| tetX | AAATTTGTTACCGACACGGAAGTT | CATAGCTGAAAAAATCCAGGACAGTT | Tetracycline |
| tnpA-01 | CATCATCGGACGGACAGAATT | GTCGGAGATGTGGGTGTAGAAAGT | Transposase |
| tnpA-02 | GGGCGGGTCGATTGAAA | GTGGGCGGGATCTGCTT | Transposase |
| tnpA-03 | AATTGATGCGGACGGCTTAA | TCACCAAACTGTTTATGGAGTCGTT | Transposase |
| tnpA-04 | CCGATCACGGAAAGCTCAAG | GGCTCGCATGACTTCGAATC | Transposase |
| tnpA-05 | GCCGCACTGTCGATTTTTATC | GCGGGATCTGCCACTTCTT | Transposase |
| tnpA-07 | GAAACCGATGCTACAATATCCAATTT | CAGCACCGTTTGCAGTGTAAG | Transposase |
| tolC-01 | GGCCGAGAACCTGATGCA | AGACTTACGCAATTCCGGGTTA | Multidrug |
| tolC-02 | CAGGCAGAGAACCTGATGCA | CGCAATTCCGGGTTGCT | Multidrug |
| tolC-03 | GCCAGGCAGAGAACCTGATG | CGCAATTCCGGGTTGCT | Multidrug |
| Tp614 | GGAAATCAACGGCATCCAGTT | CATCCATGCGCTTTTGTCTCT | Transposase |
| ttgA | ACGCCAATGCCAAACGATT | GTCACGGCGCAGCTTGA | Multidrug |
| ttgB | TCGCCCTGGATGTACACCTT | ACCATTGCCGACATCAACAAC | Multidrug |
| vanA | AAAAGGCTCTGAAAACGCAGTTAT | CGGCCGTTATCTTGTAAAAACAT | Vancomycin |
| vanB-01 | TTGTCGGCGAAGTGGATCA | AGCCTTTTTCCGGCTCGTT | Vancomycin |
| vanB-02 | CCGGTCGAGGAACGAAATC | TCCTCCTGCAAAAAAAGATCAAC | Vancomycin |
| vanC-01 | ACAGGGATTGGCTATGAACCAT | TGACTGGCGATGATTTGACTATG | Vancomycin |
| vanC-03 | AAATCAATACTATGCCGGGCTTT | CCGACCGCTGCCATCA | Vancomycin |
| vanC1 | AGGCGATAGCGGGTATTGAA | CAATCGTCAATTGCTCATTTCC | Vancomycin |
| vanC2/vanC3 | TTTGACTGTCGGTGCTTGTGA | TCAATCGTTTCAGGCAATGG | Vancomycin |
| vanG | ATTTGAATTGGCAGGTATACAGGTTA | TGATTTGTCTTTGTCCATACATAATGC | Vancomycin |
| vanHB | GAGGTTTCCGAGGCGACAA | CTCTCGGCGGCAGTCGTAT | Vancomycin |
| vanHD | GTGGCCGATTATACCGTCATG | CGCAGGTCATTCAGGCAAT | Vancomycin |
| vaSRA-01 | CCCTTACTCCCACCGAGTTTT | TTCGTCGCCCCATATCTCAT | Vancomycin |
| vaSRA-02 | CCACTCCGGCCTTGTCATT | GCTAACCACATTCCCCTTGTTTT | Vancomycin |
| vaSRB | GCCCTGTCGGATGACGAA | TTACATAGTCGTCTGCCTCTGCAT | Vancomycin |
| vaSRC | TGCGGGAAAAACTGAACGA | CCCCCCATACGGTTTTGATTA | Vancomycin |
| vaSRC4 | AGTGCTTTGGCTTATCTCGAAAA | TCCGGCAGCATCACATCTAA | Vancomycin |
| vaSRD | TTATAATGGCAAGGATGCACTAAAGT | CGTCTACATCCGGAAGCATGA | Vancomycin |
| vanSA | CGCGTCATGCTTTCAAAATTC | TCCGCAGAAAGCTCAATTTGTT | Vancomycin |
| vanSB | GCGCGGCAAATGACAAC | TTTGCCATTTTATTCGCACTGT | Vancomycin |
| vanSC-02 | GCCATCAGCGAGTCTGATGA | CAGCTGGGATCGTTTTTCCTT | Vancomycin |
| vanSE | TGGCCGAAGAAGCAGGAA | CAATAATACTCGTCAAAGGAGTTCTCA | Vancomycin |
| vanTC-01 | CACACGCATTTTTTCCCATCTAG | CAGCCAACAGATCATCAAAACAA | Vancomycin |
| vanTC-02 | ACAGTTGCCGCTGGTGAAG | CGTGGCTGGTCGATCAAAA | Vancomycin |
| vanTE | GTGGTGCCAAGGAAGTTGCT | CGTAGCCACCGCAAAAAAAT | Vancomycin |
| vanTG | CGTGTAGCCGTTCCGTTCTT | CGGCATTACAGGTATATCTGGAAA | Vancomycin |
| vanWB | CGGACAAAGATACCCCCTATAAAG | AAATAGTAAATTGCTCATCTGGCACAT | Vancomycin |
| vanWG | ACATTTTCATTTTGGCAGCTTGTAC | CCGCCATAAGAGCCTACAATCT | Vancomycin |
| vanXA | CGCTAAATATGCCACTTGGGATA | TCAAAAGCGATTCAGCCAACT | Vancomycin |
| vanXB | AGGCACAAAATCGAAGATGCTT | GGGTATGGCTCATCAATCAACTT | Vancomycin |
| vanXD | TAAACCGTGTTATGGGAACGAA | GCGATAGCCGTCCCATAAGA | Vancomycin |
| vanYB | GGCTAAAGCGGAAGCAGAAA | GATATCCACAGCAAGACCAAGCT | Vancomycin |
| vanYD-01 | AAGGCGATACCCTGACTGTCA | ATTGCCGGACGGAAGCA | Vancomycin |
| vanYD-02 | CAAACGGAAGAGAGGTCACTTACA | CGGACGGTAATAGGGACTGTTC | Vancomycin |
| vatB-01 | GGAAAAAGCAACTCCATCTCTTGA | TCCTGGCATAACAGTAACATTCTGA | MLSB |
| vatB-02 | TTGGGAAAAAGCAACTCCATCT | CAATCCACACATCATTTCCAACA | MLSB |
| vatC-01 | CGGAAATTGGGAACGATGTT | GCAATAATAGCCCCGTTTCCTA | MLSB |
| vatC-02 | CGATGTTTGGATTGGACGAGAT | GCTGCAATAATAGCCCCGTTT | MLSB |
| vatE-01 | GGTGCCATTATCGGAGCAAAT | TTGGATTGCCACCGACAAT | MLSB |
| vatE-02 | GACCGTCCTACCAGGCGTAA | TTGGATTGCCACCGACAATT | MLSB |
| vgaA-01 | CGAGTATTGTGGAAAGCAGCTAGTT | CCCGTACCGTTAGAGCCGATA | MLSB |
| vgaA-02 | GACGGGTATTGTGGAAAGCAA | TTTCCTGTACCATTAGATCCGATAATT | MLSB |
| vgb-01 | AGGGAGGGTATCCATGCAGAT | ACCAAATGCGCCCGTTT | MLSB |
| vgbB-01 | CAGCCGGATTCTGGTCCTT | TACGATCTCCATTCAATTGGGTAAA | MLSB |
| vgbB-02 | ATACGAGCTGCCTAATAAAGGATCTT | TGTGAACCACAGGGCATTATCA | MLSB |
| yceE/mdtG-01 | TGGCACAAAATATCTGGCAGTT | TTGTGTGGCGATAAGAGCATTAG | Multidrug |
| yceE/mdtG-02 | TTATCTGTTTTCTGCTCACCTTCTTTT | GCGTGGTGACAAACAGGCTTA | Multidrug |
| yceL/mdtH-01 | TCGGGATGGTGGGCAAT | CGATAACCGAGCCGATGTAGA | Multidrug |
| yceL/mdtH-02 | CGCGTGAAACCTTAAGTGCTT | AGACGGCTAAACCCCATATAGCT | Multidrug |
| yceL/mdtH-03 | CTGCCGTTAAATGGATGTATGC | ACTCCAGCGGGCGATAGG | Multidrug |
| yidY/mdtL-01 | GCAGTTGCATATCGCCTTCTC | CTTCCCGGCAAACAGCAT | Multidrug |
| yidY/mdtL-02 | TGCTGATCGGGATTCTGATTG | CAGGCGCGACGAACATAAT | Multidrug |

Table S2. Pearson correlation analysis between MGEs and classes of ARGs.

|  | Aminoglycoside | Beta_Lactamase | Chloramphenicol | MLSB | Multidrug | Others | Sulfonamide | Tetracycline | Vancomycin |
| --- | --- | --- | --- | --- | --- | --- | --- | --- | --- |
| MGEs | .785^**^ | 0.387 | 0.329 | .885^**^ | 0.167 | -0.14 | -0.0855 | .839^**^ | 0.234 |

| **. Correlation is significant at the 0.01 level (2-tailed). |
| --- |
|  |


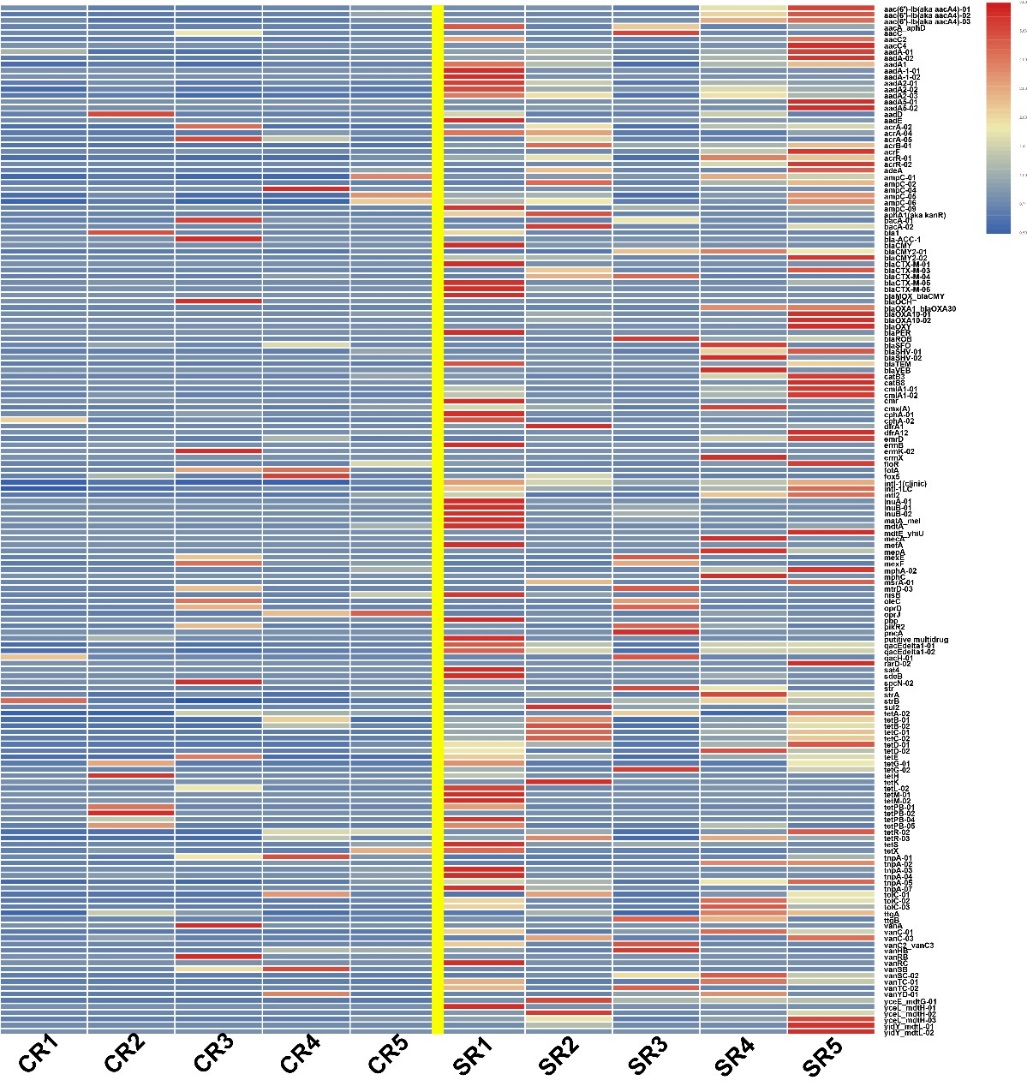


Figure S1. Heatmap analysis of ARGs and MGEs in salad samples. The order of the genes follows the classification of ARGs. CR and SR represent fast-food chain and casual dining restaurants, respectively.


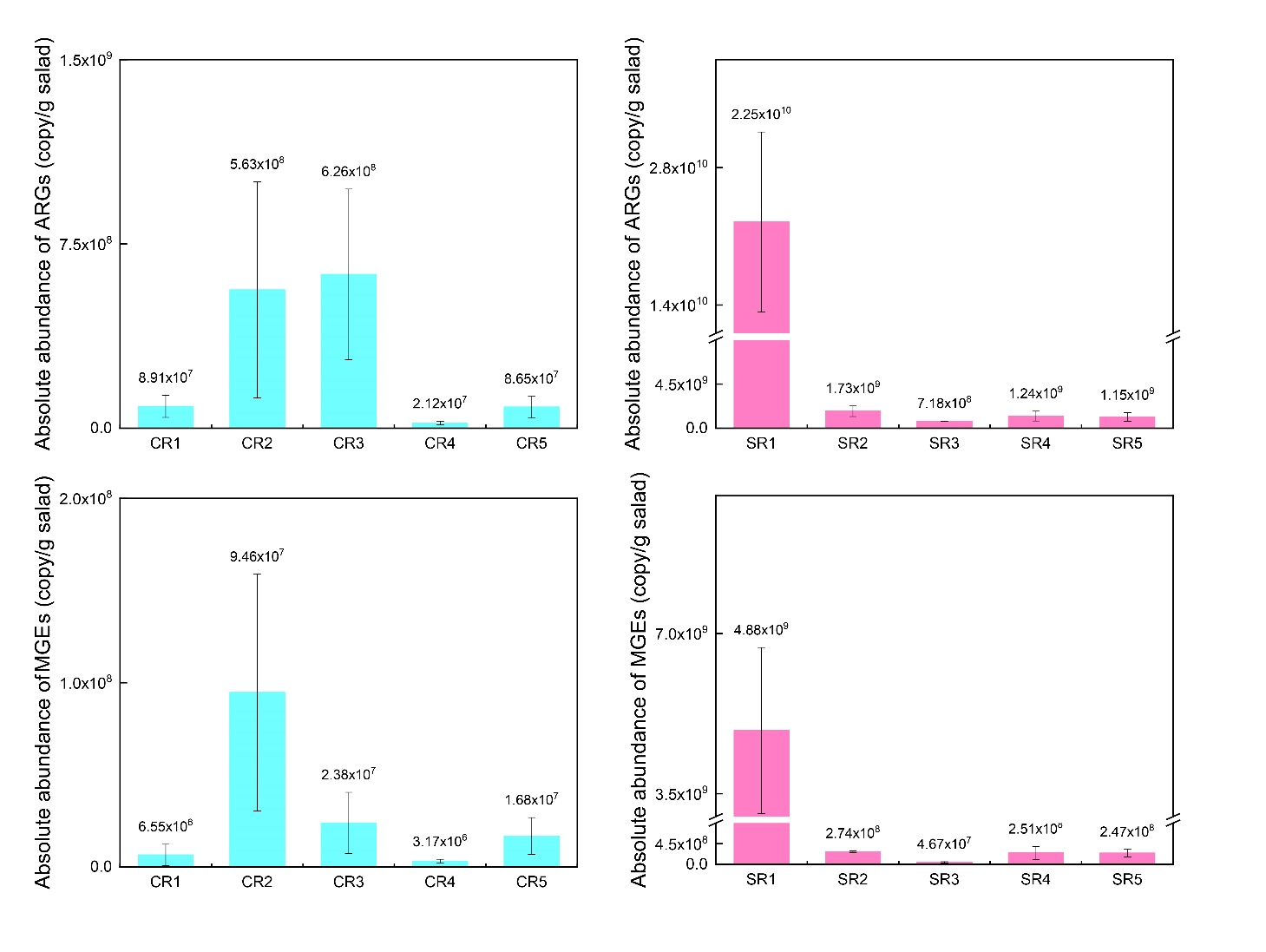


Figure S2. Absolute copy numbers of ARGs and MGEs in salad samples from fast-food chain (CR) and casual dining (SR) restaurants.


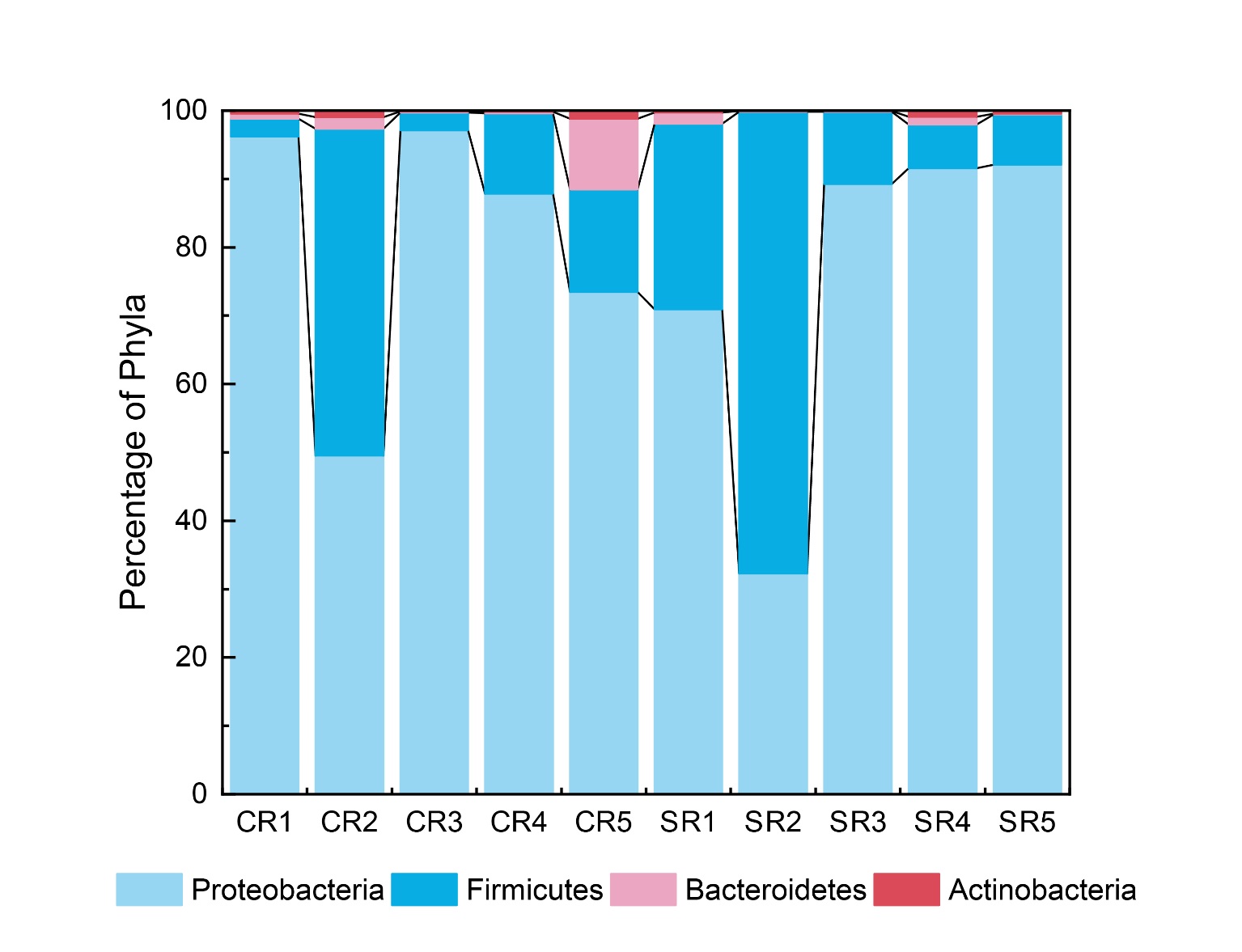


Figure S3. Percentage of detected phyla in salad samples.


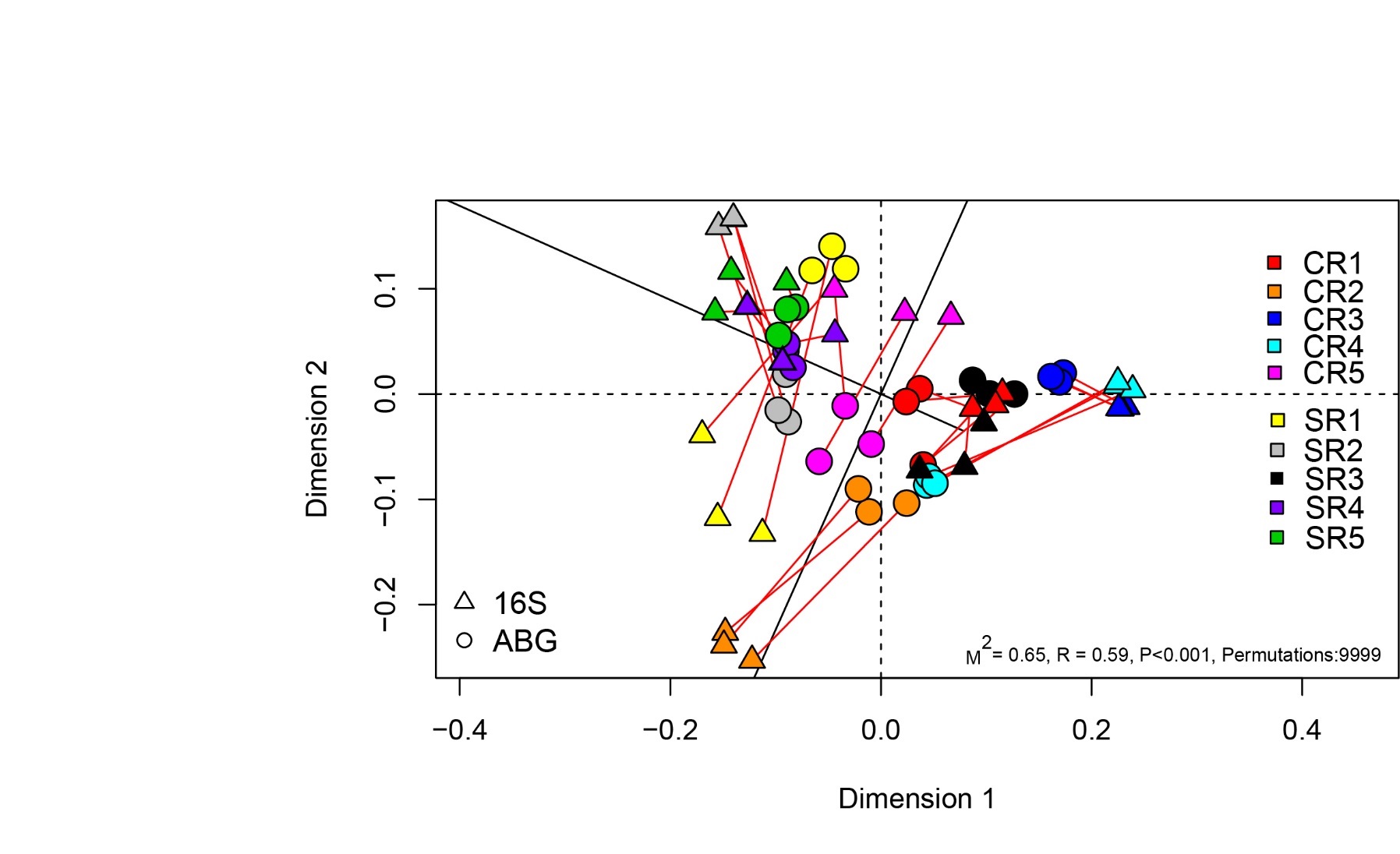


Figure S4. Procrustes analysis between ZOTUs and ARGs profiles (M^2^=0.65, R=0.59, *P* <0.05 9999 free permutations) in salad samples. Triangles and circles represent microbial communities and ARGs respectively.
